# Lomofungin and dilomofungin: inhibitors of MBNL1-CUG RNA binding with distinct cellular effects

**DOI:** 10.1093/nar/gku275

**Published:** 2014-05-05

**Authors:** Jason W. Hoskins, Leslie O. Ofori, Catherine Z. Chen, Amit Kumar, Krzysztof Sobczak, Masayuki Nakamori, Noel Southall, Samarjit Patnaik, Juan J. Marugan, Wei Zheng, Christopher P. Austin, Matthew D. Disney, Benjamin L. Miller, Charles A. Thornton

**Affiliations:** 1 Department of Neurology, University of Rochester, Rochester, NY 14642, USA; 2 Department of Chemistry, University of Rochester, Rochester, NY 14642, USA; 3 Division of Pre-clinical Innovation, National Center for Advancing Translational Sciences, National Institutes of Health, Bethesda, MD 20892, USA; 4 Department of Chemistry, Scripps Florida, Jupiter, FL 33458, USA; 5 Department of Dermatology, University of Rochester, Rochester, NY 14642, USA

## Abstract

Myotonic dystrophy type 1 (DM1) is a dominantly inherited neuromuscular disorder resulting from expression of RNA containing an expanded CUG repeat (CUG^exp^). The pathogenic RNA is retained in nuclear foci. Poly-(CUG) binding proteins in the Muscleblind-like (MBNL) family are sequestered in foci, causing misregulated alternative splicing of specific pre-mRNAs. Inhibitors of MBNL1-CUG^exp^ binding have been shown to restore splicing regulation and correct phenotypes in DM1 models. We therefore conducted a high-throughput screen to identify novel inhibitors of MBNL1-(CUG)_12_ binding. The most active compound was lomofungin, a natural antimicrobial agent. We found that lomofungin undergoes spontaneous dimerization in DMSO, producing dilomofungin, whose inhibition of MBNL1–(CUG)_12_ binding was 17-fold more potent than lomofungin itself. However, while dilomofungin displayed the desired binding characteristics *in vitro*, when applied to cells it produced a large increase of CUG^exp^ RNA in nuclear foci, owing to reduced turnover of the CUG^exp^ transcript. By comparison, the monomer did not induce CUG^exp^ accumulation in cells and was more effective at rescuing a CUG^exp^-induced splicing defect. These results support the feasibility of high-throughput screens to identify compounds targeting toxic RNA, but also demonstrate that ligands for repetitive sequences may have unexpected effects on RNA decay.

## INTRODUCTION

Myotonic dystrophy type 1 (DM1) is the prototypical genetic disease resulting from a toxic gain-of-function by mutant RNA (1). RNA toxicity has been implicated in several other disorders that share a distinctive set of genetic features: the causal mutations are large polymorphic expansions of simple tandem repeats that are genetically unstable and located in non-coding regions of the genome (2–9). The underlying disease mechanisms are heterogeneous and postulated to include activation of signalling pathways by mutant RNA, aberrant translation that initiates within the repeat tract without an AUG start codon and sequestration of proteins that bind to long tracts of CUG, CCUG, GGCCUG, GGGGCC, CGG, AUUCU or UGGAA repeats [recently reviewed by Krzyzosiak *et al.* (10)].

In DM1 the expanded sequence is a (CTG)_n_ repeat in the 3’ untranslated region (UTR) of *DM protein kinase* (*DMPK*), a gene expressed in muscle, heart and brain (11). When pathologically expanded beyond 50 repeats, the CTG tract is highly unstable in germline and somatic cells, which leads to *DMPK* alleles having several thousand repeats (12). The *DMPK* mRNA containing an expanded CUG repeat (CUG^exp^) is retained in the nucleus in foci (13,14). Splicing factors in the Muscleblind-like (MBNL) family, which are the predominant r(CUG)_n_ binding proteins in mammalian cells, are sequestered in the foci of CUG^exp^ RNA (15,16). The resulting loss of MBNL function causes misregulated alternative splicing and other changes of the muscle transcriptome (17–19). For example, mis-splicing of *insulin receptor* and *chloride ion channel 1* lead to insulin resistance and muscle hyperexcitability (myotonia), respectively (20,21).

As yet there are no disease-modifying treatments for DM1 or other RNA dominant disorders. However, several compounds or oligonucleotides have shown beneficial effects in cell or animal models (22–31). For small molecules the predominant approach has been to identify, from the repertoire of known nucleic acid binders, a set of compounds that show preferential binding to CUG repeats (22,27–29,32–34). CUG-binding compounds have also been assembled using dynamic combinatorial libraries (24,26). These studies have indicated that small molecules can improve DM1-related splicing defects by inhibiting MBNL-CUG^exp^ binding, thus restoring MBNL function in cells.

We performed a high-throughput screen to identify compounds that inhibit r(CUG)_n_ binding to MBNL1, the predominant MBNL protein of skeletal muscle. Out of 279 433 compounds in the screen, the most potent inhibitor was lomofungin, a natural antimicrobial agent from *Streptomyces lomondensis* (35,36). We found that lomofungin undergoes spontaneous dimerization in dimethyl sulfoxide (DMSO), producing dilomofungin, whose potency was 17-fold greater than lomofungin in the same screen. However, while dilomofungin displayed greater r(CUG)_n_ affinity and stronger MBNL-(CUG)_12_ binding inhibition *in vitro*, it was the monomer that showed greater correction of MBNL1-dependent splicing in cells. This discrepancy between the cell-free and intracellular activities appeared to result from a novel effect of dilomofungin on RNA turnover. Dilomofungin reduced the rate of CUG^exp^ decay in cells, which led to a striking increase of CUG^exp^ RNA in nuclear foci. These results are cautionary for development of high affinity RNA ligands to mitigate RNA toxicity, which in some cases may stabilize the target and promote the accumulation of mutant RNA in cells.

## MATERIALS AND METHODS

### High-throughput screen for inhibitors of MBNL1-CUG RNA binding

Our homogeneous time-resolved fluorescence energy transfer (HTRF) assay for inhibitors of MBNL1-(CUG)_12_ binding was previously described (37). In brief, the assay employed a chemically synthesized (CUG)_12_ oligoribonucleotide and recombinant human MBNL1 protein. The (CUG)_12_ oligonucleotide was biotinylated at the 5′ end and contained a 4 bp GC clamp at 5′ and 3′ ends to stabilize the repeat in a hairpin conformation, with G·C and C·G base pairs separated by periodic U·U mismatch in the stem. The recombinant MBNL1 had a 105 amino acid deletion at the C-terminus, to reduce protein aggregation without loss of r(CUG)_n_ binding, a GST tag at the N-terminus and a His_6_ tag at the C-terminus. The MBNL1-Δ105-His_6_ was expressed and purified from *Escherichia coli* as previously described (37). The biotin-(CUG)_12_ RNA and MBNL1-Δ105-His_6_ protein were mixed in equimolar concentrations (20 nM each) and dispensed in 1536 well plates at 2 μl per well. Compounds dissolved in DMSO were then added (23 nl per well) in a five point dilution series that ranged from 92 nM to 57.5 μM. All assay buffers contained 0.05% Tween-20 to reduce aggregation effects (38). After 15 min incubation at room temperature, detection reagents were added and HTRF activity was determined on a plate reader (EnVision PerkinElmer, Boston, MA, USA). In the primary screen the fluorescence donor was terbium conjugated to anti-His_6_ antibody that bound to MBNL1-Δ105-His_6_, and the fluorescence acceptor was XL665 conjugated to streptavidin that bound to biotin-(CUG)_12_. The confirmatory assay employed a different detection system, consisting of AlphaScreen donor beads (conjugated to streptavidin) that emitted a singlet oxygen to activate AlphaScreen acceptor beads (coated with nickel, PerkinElmer). Both systems were shown to detect MBNL1-Δ105-His_6_ when in close proximity to biotin-CUG_12_ in solution (37). The assay was used to screen 279 433 compounds in the Molecular Libraries Small Molecule Repository (MLSMR, Supplementary Table S1). A description of the library and selection algorithm for the compounds can be found at http://mli.nih.gov/mli/compound-repository/mlsmr-compounds/. Notably, the selection algorithm was not designed to enrich for RNA binding compounds.

### Identification and structural analysis of dilomofungin

Analysis of lomofungin (Enzo Life Sciences, BML-A245–0050) was performed on a Shimadzu LC2010 HPLC equipped with a C18 reverse-phase column. ^1^H NMR spectra were recorded at 25°C on a Bruker Avance 400 (400 MHz) or Bruker Avance 500 (500 MHz) instrument. Chemical shifts (δ) are reported in parts per million (ppm) downfield from tetramethylsilane and referenced to the residual protium signal in the nuclear magnetic resonance (NMR) solvent (CDCl_3_, δ = 7.26). Data are reported as chemical shift, multiplicity (s = singlet, d = doublet, t = triplet, m = multiplet), integration, and coupling constant (J) in Hertz (Hz). ^13^C NMR spectra were likewise recorded at 25°C on a Bruker Avance 400 (100 MHz) or Bruker Avance 500 (125 MHz). Chemical shifts (δ) are reported in parts per million (ppm) downfield from tetramethylsilane and referenced to carbon resonances in the NMR solvent. High-resolution mass spectra were acquired at the University of Buffalo mass spectrometry facility, Buffalo, NY, USA.

### Fluorescence titration

Fluorescence titrations were performed using a Varian Cary Eclipse spectrophotometer. A 50 μM stock of compound in 1X Hepes buffered saline with 0.5% DMSO and 0.005% tween-20 was titrated into 400 μl of Cy3- labeled (CUG)_10_ RNA, also in the same buffer. After each addition the mixture was allowed to equilibrate for at least 10 min, or until no change in the fluorescence spectrum was observed. Changes in fluorescence emission at 565 nm (excitation at 550 nm) were measured. Raw data were corrected for dilution-dependent changes, plotted against compound concentration and fit to the one site binding equation (Equation 1):

(1) \begin{equation*}
y = (b_{\max } \times x)/(K_{\rm D} + x)
\end{equation*}

### Competition dialysis

Synthetic oligonucleotides (Integrated DNA Technologies, Inc., Coralville, IA, USA) were amplified by polymerase chain reaction (PCR) as previously described (28,32). The PCR products were used as templates for *in vitro* transcription (RNAMaxx kit, Stratagene). The transcripts were purified on denaturing 10% polyacrylamide (19:1) gels. The RNA was visualized by UV shadowing, excised, eluted in 0.3 M NaCl and precipitated in three volumes of ethanol overnight at −80°C. The samples were resuspended in 10 mM phosphate buffer (pH 7.2). The RNA concentration was determined by absorbance at 260 nm using the corresponding extinction coefficients. Extinction coefficients were determined using HyTher version 1.0 (39,40) based on nearest neighbor analysis (41).

A fresh 3.27 mM stock solution of lomofungin was prepared in 500 μl of DMSO and stored at −20°C. An aliquot of this stock solution was dissolved in 200 ml of phosphate buffer (8 mM NaH_2_PO_4_ and Na_2_HPO_4_ 110 mM KCl, 2 mM MgCl_2_, 2 mM CaCl_2_ and 1 mM Na_2_EDTA at pH 7.0). A similar procedure was used to prepare a 10 mM stock solution of dilomofungin. Slide-A-Lyzer MINI dialysis units (Pierce Biotechnology, Rockford, IL, USA) were used for competition dialysis assays. Each unit was evaluated before use by dialyzing against water for 24 h. The volume in each unit was measured, and units with volumes exceeding the original 200 μl were discarded.

Competition dialysis was performed as previously described (42). Briefly, dialysis units containing 0.1 ml of 1.56 μM full length RNA were placed into 200 ml of a 1 μM ligand solution in phosphate buffer. The samples were allowed to equilibrate with the dialysate by stirring at 200 rpm for 24 h at room temperature (20–22°C). Previous experiments have shown that this interval is sufficient for samples to reach equilibrium (42,43). For experiments to compare ligand binding to MBNL1 protein with RNA hairpins containing incremental numbers of 5′CUG/3′GUC motifs, 1.33 μM of each biomolecule was placed in the dialysis units.

At the end of the equilibration period, 67.5 μl of each sample was carefully removed from the dialysis unit and transferred to a microcentrifuge tube. To each sample, 7.5 μl of 10% (w/v) sodium dodecyl sulfate (SDS) was added, in order to dissociate the lomofungin or dilomofungin from nucleic acid or MBNL1. The SDS step was completed to ensure accurate determination of the concentration of the bound ligand, as the spectroscopic properties of bound ligands could differ from unbound ligands.

The total ligand concentration (*C_t_*) within each dialysis unit was determined spectrophotometrically. Appropriate corrections were made for the small dilution resulting from the addition of the SDS stock solution. In parallel the free ligand concentration (*C_f_*) was determined from an aliquot of the dialysate solution, which typically did not vary appreciable from the initial 1 μM concentration. Absorbance and fluorescence measurements were made using a UV-Visible spectrophotometer (Beckman Coulter, DU 800). The bound ligand concentration (*C_b_*) was then determined by using Equation (2):

(2) \begin{equation*}
C_b = C_t - C_f
\end{equation*}

Pairwise signed fold changes were calculated from the bound ligand concentrations (C*_b_*). *P*-values for differential ligand binding were determined by unpaired *t*-tests, and α significance thresholds were false discovery rate (FDR) controlled for multiple testing.

### Luciferase assay

The fluc0 and fluc800 C2C12 lines (also known as C1-S and C5–14) that express firefly luciferase fused to the *DMPK* 3′ untranslated region with 0 or 800 CTG repeats were previously described (23). Assays for luciferase activity were performed as previously described (26), 3 days after addition of vehicle or drug.

### Fluorescence *in situ* hybridization and immunofluorescence

Fluorescence *in situ* hybridization (FISH) for CUG repeat RNA and immunofluorescence for MBNL1 were performed as previously described (30) 3 days after addition of vehicle or drug.

### Quantitative and semi-quantitative RT-PCR

TaqMan qRT-PCR analysis of *fluc0* and *fluc800* transcript levels was performed using a primer probe set that spanned a chimeric intron in the 5′ UTR of the transgene.

Probe: 5′ /56-FAM/CACAATAACCAGCACGTTGCCCA/3IABkFQ/ 3′

Primer 1: 5′ GACTGACCGCGTTACTCC 3′

Primer 2: 5′ AGAATAGGAACTTCGGAATAGGAAC 3′

The *fluc* transcripts were quantitated using the ΔΔCt method with GAPDH (ABI, Cat# 4352339E) as endogenous control. Semi-quantitative RT-PCRs were also performed for *Mapkapk5*, *Arid2*, *Nr3c1*, *Mapkap1* and rRNA 5′ external transcribed spacer (ETS) using primers listed in Supplementary Table S6. RNA was extracted 3 days after addition of vehicle or drug.

### Conditional cell model for analysis of MBNL1 splicing regulatory activity

Plasmid pLC16 for conditional transcription of 800 CTG repeats was previously described (44). To establish a cell system for splicing analysis, we stably transfected C2C12 mouse muscle cells with pLC16. PhiC31 integrase was co-expressed to produce single copy integrations. Transfection was performed using Nucleofector (Lonza) according to the manufacturer's program B-32. After stably transfected cells were selected in puromycin, transcription across the expanded repeat was activated by Cre-mediated excision of a transcription terminator cassette as previously described (44). For MBNL1 knockdown, cells were transfected with ON TARGET-plus MBNL1-targeting siRNA (Dharmacon) using Lipofectamine RNAiMAX (Invitrogen). For antisense inhibition of CUG^exp^-MBNL1 binding we nucleofected cells with 2 μM of CAG25, a 25-mer morpholino oligonucleotide comprised of CAG repeats (30). For RT-PCR analysis of *Serca1* alternative splicing the RNA was harvested and cDNA was synthesized as described previously (44). PCR amplification was carried out for 22 cycles using primers 5′-GCTCATGGTCCTCAAGATCTCAC-3′ and 5′-GGGTCAGTGCCTCAGCTTTG-3′, flanking the alternative exon 22. PCR products were separated on agarose gels, stained with SYBR Green I (Invitrogen), scanned with a laser fluorimager (Typhoon, GE Healthcare) and quantified using ImageQuant as previously described (16).

### RNA decay analysis

Actinomycin D (Sigma) was added to the media of fluc0 (no repeat) or fluc800 (800 CTG repeats) cells for up to 8 h in the presence or absence of 10 μM of dilomofungin. Levels of transgene mRNA were determined by qRT-PCR as previously described (44), normalized to18S rRNA.

## RESULTS

### High-throughput screen

**Figure 1.**
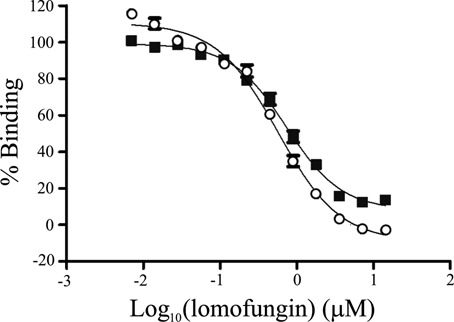
HTRF (black squares) and AlphaScreen (open circles) assays showed inhibition of biotin-CUG_12_ binding to MBNL1-Δ105-His_6_ protein by lomofungin. Subsequent analyses indicated two molecular species in the lomofungin stock. Error bars represent 1 s.d.

A screen for inhibitors of MBNL1-(CUG)_12_ binding was performed on 279 433 compounds in the MLSMR (Supplementary Table S1). The primary screen employed recombinant MBNL1 and biotinylated (CUG)_12_ in a solution-phase HTRF assay (PubChem AID 2675). Each compound was tested in a five-point 1:5 dilution series ranging from 92 nM to 57.5 μM, and concentration-response curve fitting was performed (37,45). The screen identified 140 compounds (0.05%) that showed ≥ 60% inhibition at 57 μM, excluding compounds having a biotin-like structure. Of these, 135 were available as DMSO solutions and were selected for confirmatory and counter screens. These included (i) a repeat of the initial HTRF assay (Pubchem AID 493199); (ii) an orthogonal confirmatory assay of the same MBNL1-(CUG)_12_ binding event using a different detection method (AlphaScreen assay, PubChem AID 493205); and (iii) a counter screen using an HTRF protein–peptide binding assay (Pubchem AID 651843). Ten compounds showed consistent inhibitory activity in the confirmatory and orthogonal assays but not in the counter assay, and were ordered as powder samples for retesting in the AlphaScreen (Supplementary Figure S1). Among these, the most potent inhibitor was the antimicrobial agent lomofungin, which showed an IC_50_ more than 2-fold lower than other compounds in the screen (Figure 1). However, quality control analysis revealed the presence of two species in the lomofungin stock.

### Lomofungin dimerizes in DMSO, producing a stronger inhibitor of MBNL1-CUG RNA binding

**Figure 2.**
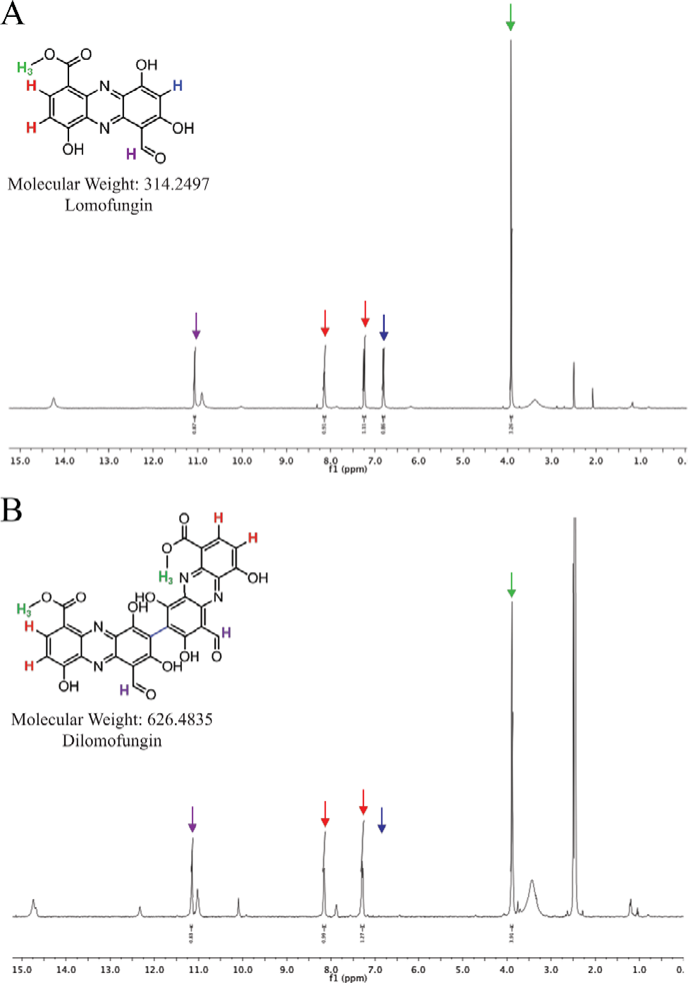
^1^H-NMR indicates structure of dimerized lomofungin. (**A**) Lomofungin ^1^H NMR (500 MHz, DMSO-d6) ppm: 11.05 (s, 1H), 8.13 (d, *J* = 7.7 Hz, 1H), 7.24 (d, J = 7.9 Hz, 1H), 6.80 (s, 1H), 3.92 (s, 3H). (**B**) Dilomofungin ^1^H NMR (500 MHz, DMSO-d6) δ 11.20 (s, 2H), 8.20 (d, *J* = 8.0 Hz, 2H), 7.33 (d, *J* = 8.0 Hz, 2H), 3.93 (s, 6H). Colored arrows indicate peaks for the corresponding hydrogens in the lomofungin and dilomofungin structures.

HPLC analysis of lomofungin that was freshly prepared in DMSO showed a single peak with a retention time that was markedly different from a previously prepared solution (Supplementary Figure S2A). Mass spectrometry analysis of the unknown compound in the older solution indicated a mass of 626.2, two AMUs less than twice the molecular weight of lomofungin, suggesting dimerization with loss of two protons (Supplementary Figure S2B). Infrared and ^1^H- and ^13^C-NMR spectra of the unknown compound confirmed this expectation. The structure of the dimer, which we designated dilomofungin, featured a carbon–carbon bond between the same aromatic carbon on two lomofungin monomers (Figure 2A and B, and Supplementary Figure S3). A limited examination of conditions under which lomofungin dimerizes in DMSO suggested that dimerization occurs at 4°C or above and proceeds more rapidly at higher temperatures and concentrations. For example, ^1^H-NMR analysis of 10 mM lomofungin in deuterated DMSO solution showed that nearly all lomofungin was dimerized after 6 days at room temperature, and by 30 days the monomer was undetectable (Supplementary Figure S4).

**Figure 3.**
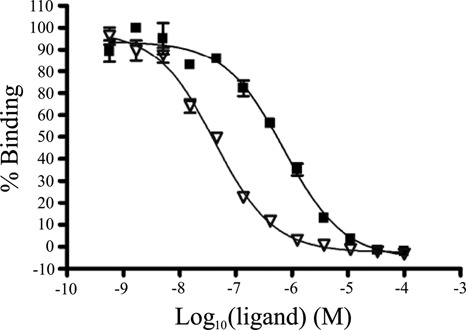
AlphaScreen assay showing that dilomofungin (open triangles) is 17-fold more potent as inhibitor of MBNL1-(CUG)_12_ binding than lomofungin (closed squares) [IC_50_s = 42 nM, 95% CI 33 to 53 nM, and 717 nM, 95% CI 533 to 964 nM, respectively].

The affinities of lomofungin and dilomofungin for Cy3-(CUG)_10_ RNA were determined by fluorescence titration (Supplementary Figure S5). The change in emission at 565 nm was plotted against drug concentration, and then fitted to a one binding site equation. The calculated *K*_D_s were 998 ± 180 nM and 606 ± 300 nM for lomofungin and dilomofungin, respectively. To compare their efficiency for inhibiting MBNL1-(CUG)_12_ binding, we repeated the AlphaScreen assay using the monomer or fully converted dimer. Dilomofungin exhibited 17-fold stronger inhibition than lomofungin, with IC_50_s of 42 nM (95% CI, 33–53 nM) and 717 nM (95% CI, 533–964 nM), respectively (Figure 3).

### RNA binding properties of lomofungin and dilomofungin

**Figure 4.**
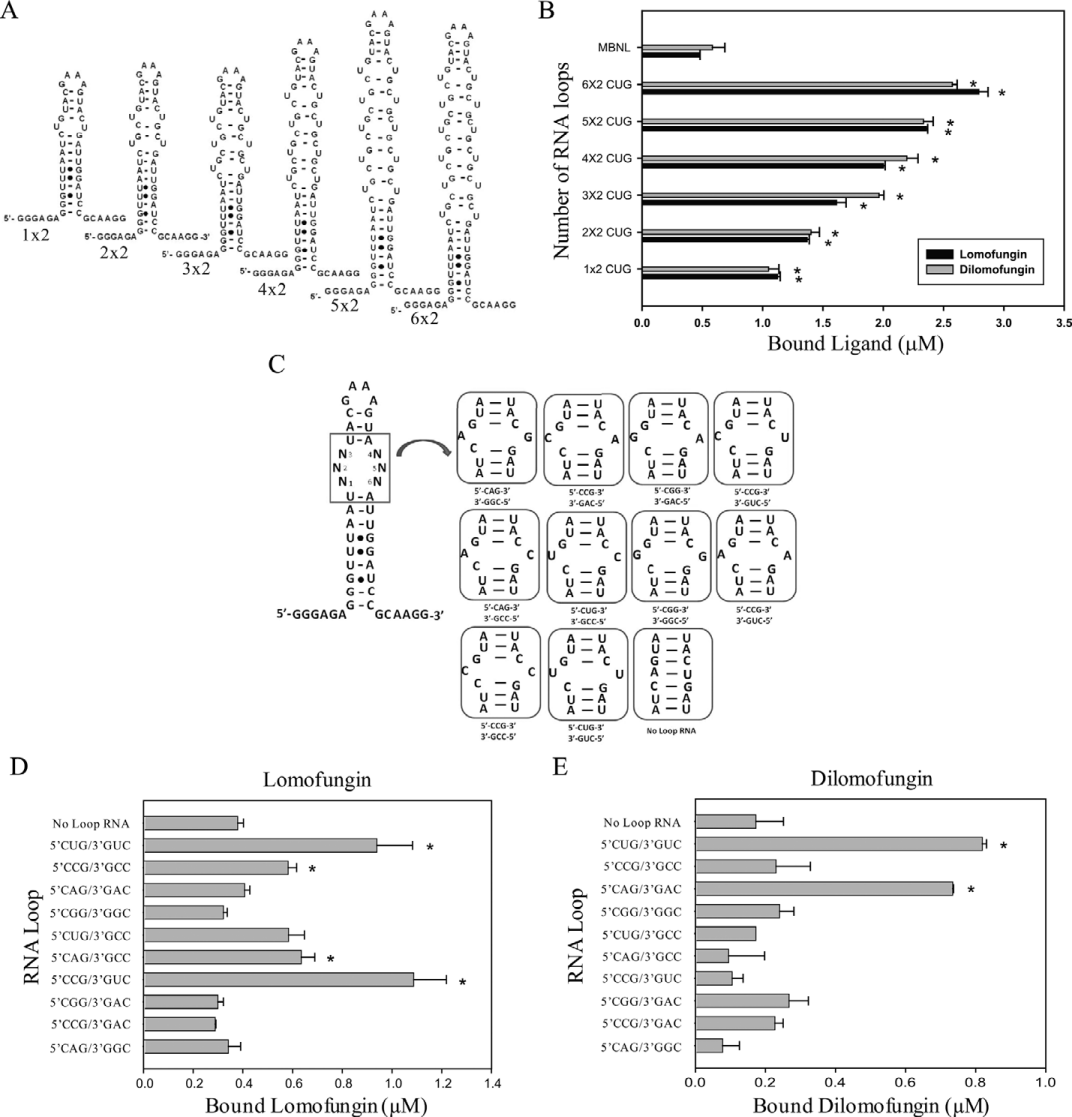
Competition dialysis indicated that lomofungin and dilomofungin bind to 5′CUG/3′GUC internal loops in preference to MBNL1 protein or perfectly duplexed RNA. (**A**) Diagram of hairpin RNAs that contained increasing numbers of 5′CUG/3′GUC internal loops. (**B**) The amount of lomofungin or dilomofungin bound to MBNL1 protein or the indicated RNA during competition dialysis. * indicates *P*_adjusted_ < 0.05 for comparison of indicated RNA versus MBNL1 binding. (**C**) Diagram of RNA hairpins with single nucleotide internal loops used for competition dialysis. (**D, E**) The amount of lomofungin (D) or dilomofungin (E) bound to RNAs displaying the indicated single nucleotide internal loops or ‘no loop RNA’. * denotes differential binding of compound to the indicated RNA hairpin as compared to ‘no loop RNA’, *P*_adjusted_ < 0.05. All error bars represent 1 s.d.

We used competition dialysis to compare the relative binding of lomofungin and dilomofungin to different targets. First, we examined binding to RNA hairpins containing 1–6 consecutive 5′CUG/3′GUC internal loops (Figure 4A) as compared to MBNL1-Δ105-His_6_ protein. Both lomofungin and dilomofungin showed a > 1.5-fold binding preference for RNA with a single internal 5′CUG/3′GUC loop, as compared to MBNL1 protein (*P* = 0.005 and 0.023, respectively). The binding preference for CUG repeats versus MBNL1 protein was increased to > 4-fold for RNA with six internal 5′CUG/3′GUC loops (*P* = 0.0044 and 0.0008, respectively) (Figure 4B and Supplementary Tables S2 and S3). Notably, while lomofungin and dilomofungin both bound at 1:1 stoichiometry to RNA with a single 5′CUG/3′GUC internal loop, the increase in compound binding to RNAs containing additional 5′CUG/3′GUC motifs was not proportional to the number of repeats, suggesting negative cooperativity in binding to adjacent internal loops. The RNA with six 5′CUG/3′GUC internal loops bound to lomofungin and dilomofungin with roughly 1:2 (drug:internal loop) stoichiometry.

Next, we used competition dialysis to examine lomofungin and dilomofungin binding to a panel of 10 RNA hairpins that were identical except for a single mismatch in a 5′CNG/3′GNC internal loop. Every possible mismatch except G·U and U·G wobble pairs was tested (Figure 4C). A hairpin with perfect complementarity in the stem (‘no loop RNA’) served as control for binding to dsRNA. Dilomofungin showed modest preference for binding to the U·U mismatch as compared to no loop RNA or other possible mismatches (*P* < 0.01) except A·A (Figure 4D and E, and Supplementary Tables S4 and S5). Binding characteristics of lomofungin were broader but still showed preferential binding to U·U and other pyrimidine mismatches, as compared to purine–purine or purine–pyrimidine mismatches.

### Dilomofungin reduces decay of CUG^exp^-containing transcripts

**Figure 5.**
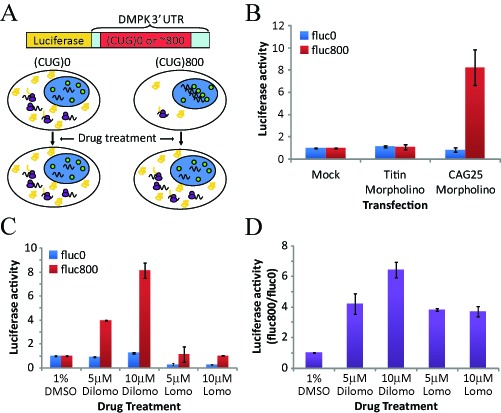
Lomofungin and dilomofungin increased luciferase activity in cells that express *firefly luciferase* fused to the *DMPK* 3′ UTR containing 800 CUG repeats. (**A**) Diagram of the luciferase assay. The stably transfected C2C12 cells expressed *firefly luciferase* (*fluc*) with 800 or 0 CUG repeats in the 3′ UTR (designated fluc800 or fluc0 cells, expressing *fluc*-CUG^exp^ or *fluc*-CUG^0^ mRNAs, respectively). Mbnl1 protein (green circles) is sequestered by *fluc*-CUG^exp^ mRNA in nuclear foci. The translation of *fluc*-CUG^exp^ mRNA is reduced by nuclear retention. When protein interactions of CUG repeats are blocked, the nucleocytoplasmic transport and translation of *fluc*-CUG^exp^ mRNA is increased. (**B**) Validation of assay using CAG25 antisense morpholino, a known binder of CUG repeats. Two days after transfection of CAG25, luciferase was upregulated in fluc800 but not fluc0 cells. Luciferase activity was normalized to mock transfected controls. Luciferase was not affected by control antisense morpholino directed against an irrelevant target (exon 362 of *Titin*). (**C**) Luciferase activity in fluc0 and fluc800 cells treated with lomofungin or dilomofungin for 3 days, normalized to cells treated with vehicle alone (1% DMSO). (**D**) Luciferase activity in lomofungin- or dilomofungin-treated cells, expressed as a ratio of fluc800 to fluc0 cells, and normalized to the ratio in vehicle-treated cells (same experiments as ‘C’). Error bars indicate 1 s.d., and * denotes *P* < 0.05.

**Figure 6.**
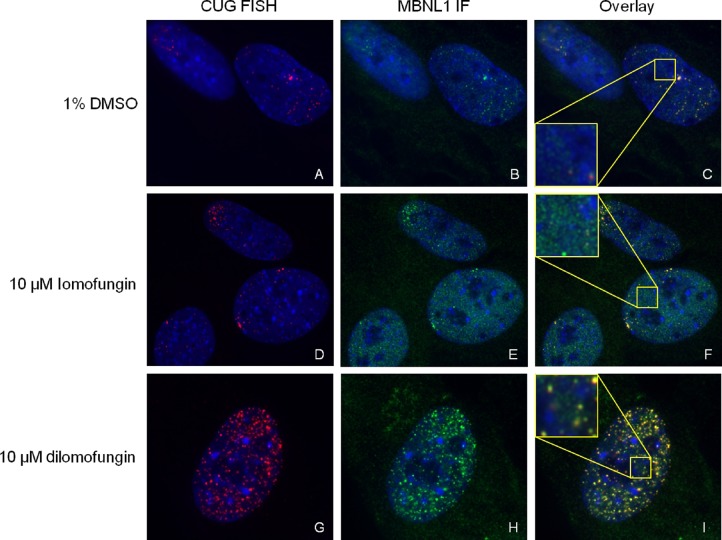
FISH combined with immunofluorescence showed increased nuclear foci of CUG^exp^ RNA in fluc800 cells treated with dilomofungin, and increased diffuse nucleoplasmic MBNL1 in cells treated with lomofungin. (**A, D** and **G**) FISH for CUG^exp^ RNA (red). (**B, E** and **H**) Immunofluorescence for MBNL1 protein (green). (**C, F, I**) Merged images. Dapi shows nuclei in each panel (blue). FISH and immunofluorescence signals are thresholded and displayed with identical settings for each column. Insets in C, F and I represent 3× magnification of the indicated region.

Previously we found that a 25-mer morpholino antisense oligonucleotide comprised of CAG repeats (‘CAG25’) was able to compete with MBNL1 for binding to CUG repeat RNA, thus disrupting MBNL1-CUG^exp^ complexes (30). Injection of CAG25 into muscle of transgenic mice produced a local increase of nuclear export and translation of CUG^exp^ mRNA, indicating that nuclear retention is overcome by masking the repeat sequence. To adapt this phenomenon for cell culture, we used C2C12 myogenic cells that were stably transfected with a construct expressing firefly luciferase (*fluc*). The *fluc* cDNA was fused to a *DMPK* 3′ UTR that contained 0 or 800 CTG repeats (fluc0 or fluc800 cells, diagrammed in Figure 5A). As expected, the fluc800 cells exhibited nuclear foci of CUG^exp^ RNA and MBNL1 protein (Figure 6), and nucleofection of CAG25 produced an 8-fold increase of luciferase activity, as compared to controls that were mock transfected or transfected with an irrelevant morpholino (Figure 5B). In contrast, nucleofection of CAG25 had no effect on luciferase expression in fluc0 cells. FISH and immunofluorescence indicated that transfection of CAG25 did not fully eliminate the nuclear CUG^exp^ foci in fluc800 cells, but did appear to increase the amount of MBNL1 staining in the nucleoplasm that was not associated with foci (Supplementary Figure S6). These results are consistent with previous *in vivo* observations that CAG25 injection produced partial release of CUG^exp^ RNA and MBNL1 protein from nuclear foci, resulting in increased MBNL1 activity and increased nuclear export and translation of CUG^exp^ mRNA (30). Notably, CAG25 produced a modest reduction of *fluc*-CUG^exp^ transcripts in both systems ((30) and data not shown), suggesting that increased nuclear export may also enhance the turnover of CUG^exp^ RNA.

Next we used fluc800 cells to examine the effects of lomofungin and dilomofungin on translation of the *fluc*-CUG^exp^ mRNA. Three days after addition to the culture media, dilomofungin produced a dose-dependent increase of luciferase activity, reaching 8.1-fold (95% CI ± 0.7) up-regulation at 10 μM (normalized to cells treated with vehicle alone, Figure 5C). In contrast, dilomofungin did not affect luciferase in fluc0 cells, and lomofungin did not increase luciferase in fluc800 cells. However, lomofungin showed cytotoxicity and luciferase inhibition at this concentration (Figure 5C and Supplementary Figure S7), consistent with previous observations that at high concentration it inhibits transcription and translation in yeast (46). When normalized to results in fluc0 cells, lomofungin also produced a modest increase of luciferase in fluc800 cells (3.7-fold at 10 μM, 95% CI ± 0.4, Figure 5D).

**Figure 7.**
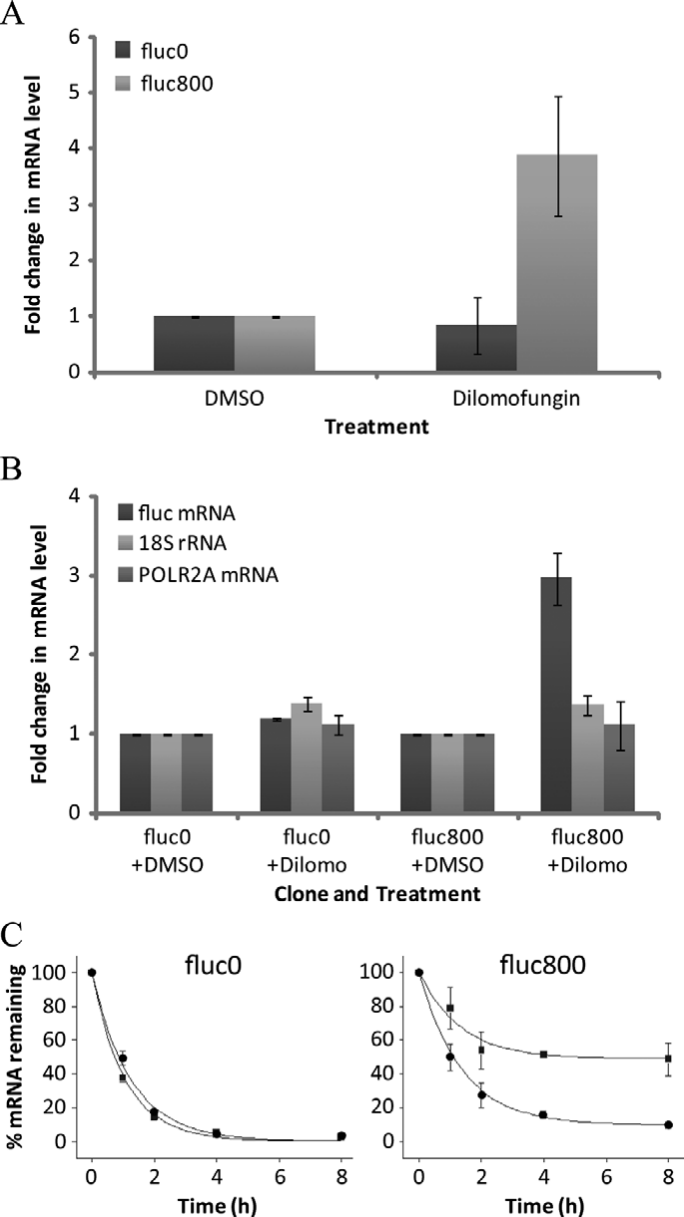
Dilomofungin stabilizes *fluc-CUG*^exp^, but not *fluc-CUG^0^* mRNA. (**A**) Levels of *fluc* mRNA in fluc0 and fluc800 cells treated with 10 μM dilomofungin or vehicle (DMSO) for 3 days were determined by qRT-PCR. Results were normalized to *Gapdh* and expressed as the fold-change relative to vehicle alone. Note that *Gapdh* C_t_ values did not vary with dilomofungin treatment. (**B**) Expression levels of *fluc*, *RNA polymerase II polypeptide A* (*Polr2a*) and *18S* rRNA in fluc0 and fluc800 cells treated with 10 μM dilomofungin or vehicle alone for 3 days. Expression levels were determined by qRT-PCR normalized to *Gapdh*. (**C**) Decay of *fluc* mRNA after addition of actinomycin D is shown for dilomofungin (10 μM) or vehicle (DMSO) treated fluc0 and fluc800 cells. *Fluc* mRNA levels were determined by qRT-PCR and normalized to 18S rRNA. The 0 h time point was set to 100% expression. Error bars indicate 1 s.d., and * denotes *P* < 0.05.

These observations raised the possibility that dilomofungin, like CAG25 morpholino, can promote the nuclear export and translation of *fluc*-CUG^exp^ transcripts. Alternatively, it was possible that the increase of luciferase resulted from greater overall accumulation of the *fluc*-CUG^exp^ mRNA, without affecting nuclear export efficiency. To distinguish these possibilities, we used FISH to examine nuclear levels of *fluc*-CUG^exp^ mRNA. Three days after addition to the culture media, dilomofungin (10 μM) but not vehicle (DMSO) produced a striking increase in the number and intensity of nuclear foci in fluc800 cells (Figure 6G), suggesting a major build-up of *fluc*-CUG^exp^ mRNA. Quantitative RT-PCR confirmed a 4-fold increase of *fluc* mRNA in dilomofungin-treated fluc800 cells (Figure 7A). By contrast, lomofungin did not affect CUG^exp^ foci (Figure 6D). To assess specificity, we used quantitative or semi-quantitative RT-PCR to examine housekeeping genes *Polr2a*, 18S rRNA, and four endogenous mRNAs that contained short runs of CNG repeats (*Mapkap1*, *Mapkapk5*, *Arid2* and *Nr3c1*, containing 25 CUG, 10 CCG, 11 CCG and 17 CAG repeats, respectively) (Figure 7B and Supplementary Figure S8). None of these transcripts showed a noticeable increase in response to dilomofungin, which suggested that the transcript accumulation induced by dilomofungin was relatively selective for *fluc*-CUG^exp^ mRNA.

To address the mechanism for CUG^exp^ accumulation, we assessed the decay of *fluc* mRNA. The transcription inhibitor actinomycin D (5 μg/ml) was applied to fluc800 or fluc0 cells, in the presence or absence of dilomofungin. The rate of *fluc800* mRNA decay was markedly reduced by dilomofungin (10 μM), producing a 5-fold increase in the calculated half-life (Figure 7C). In contrast, dilomofungin had no effect on stability of *fluc0* mRNA, or on levels of the 5′ ETS of rRNA (Supplementary Figure S7), an rRNA fragment that is generated and rapidly degraded in the nucleus (47). These results suggested that stabilization of *fluc*-CUG^exp^ mRNA by dilomofungin did not result from global inhibition of nuclear or cytoplasmic RNA decay.

**Figure 8.**
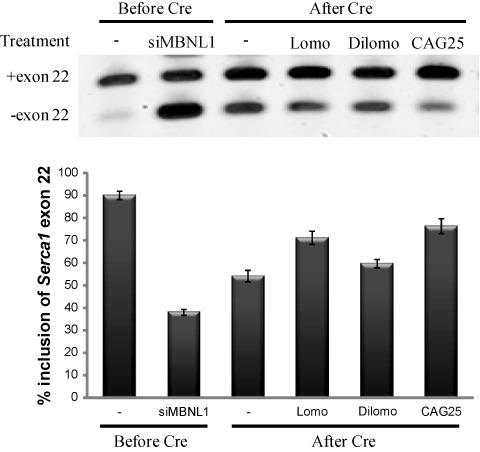
Partial rescue of *Serca1* alternative splicing by CAG25, lomofungin and dilomofungin in C2C12 cells that were stably transfected with a construct for conditional expression of CUG^exp^ RNA. Expression of (CUG)_800_ was triggered by *Cre* excision of a transcription terminator cassette. The upper panel shows representative gel images of RT-PCR assay for inclusion of *Serca1* exon 22. The lower panel indicates the combined results of three experiments. Exon 22 inclusion was reduced by siRNA knockdown of MBNL1 (siMBNL1) or Cre-mediated activation of (CUG)_800_ expression. Treatment of the (CUG)_800_ expressing cells with 10 μM lomofungin or dilomofungin, or by transfection of CAG25 morpholino, caused partial rescue of exon 22 splicing (*P* = 0.001, 0.031 and 0.007, respectively, relative to vehicle treated cells after Cre). Error bars indicate 1 s.d.

### Lomofungin improves MBNL1 splicing regulatory activity in CUG^exp^-expressing cells

Addition of lomofungin (10 μM) to the culture media led to increased diffuse nucleoplasmic staining for MBNL1 in fluc800 cells, consistent with partial alleviation of the MBNL1 sequestration (Figure 6C, F). Interestingly, despite the increase of CUG^exp^ RNA, the immunofluorescence also appeared to show that some MBNL1 protein was not associated with RNA foci in dilomofungin-treated cells (Figure 6I). To test for effects on MBNL1 splicing regulatory activity, we examined *sarco/endoplasmic reticulum Ca^2+^-ATPase 1* (*Serca1*). The alternative splicing of *Serca1* exon 22 (*Serca1*ex22) is highly conserved and strongly promoted by MBNL1 (48). Among numerous exons regulated by MBNL1, *Serca1*ex22 is perhaps the most sensitive and specific known indicator of MBNL1 activity, because it shows the largest effect in DM1 that is conserved in MBNL1 knockout mice and reversible by CUG^exp^ knockdown (16,18,31). To clearly delineate the effects of CUG^exp^ RNA on splicing regulation, we developed a cell model for conditional expression of expanded CUG repeats, again using C2C12 murine myogenic cells. C2C12 cells were stably transfected with a construct, pLC16, for conditional expression of expanded CUG repeats (44). As the cells were initially derived, the transcription of the repeat was blocked by a floxed transcription terminator element (TTE). The TTE was located upstream of a hygromycin selectable marker that was fused to the *DMPK* 3′ UTR containing 800 CUG repeats. Prior to recombination the cells were hygromycin sensitive, did not exhibit CUG^exp^ foci and showed 90 ± 2% inclusion of *Serca1*ex22 (assessed by RT-PCR, Figure 8), equivalent to non-transfected cells. In non-recombined cells the transfection of *Mbnl1* siRNA reduced the inclusion of *Serca1*ex22 to 38 ± 1%, confirming that *Serca1*ex22 is MBNL1 regulable in C2C12 cells (48). Transfection of plasmid encoding *Cre* recombinase followed by selection in hygromycin produced a population of cells that expressed CUG^exp^ RNA and displayed nuclear foci (not shown). In recombined cells the level of *Serca1*ex22 inclusion was reduced to 54 ± 3% (Figure 8), consistent with MBNL1 sequestration. Transfection of CAG25 increased the inclusion frequency back to 77 ± 4% (*P* = 0.001), consistent with partial release of sequestered MBNL1. Application of 10 μM lomofungin also promoted the inclusion of *Serca1*ex22 (71 ± 3%, *P* = 0.031, Figure 8). By comparison, dilomofungin caused a smaller but still significant increase of *Serca1*ex22 inclusion (60 ± 2%, *P* = 0.007, see the Discussion).

## DISCUSSION

Targeting of RNA with small molecules has been advocated as a therapeutic approach, especially when viable protein targets are lacking [reviewed by Tor (49) and Thomas and Hergenrother (50)]. The utility of RNA targeting is best illustrated by numerous antibiotic drugs that inhibit bacterial protein synthesis. Among well-studied examples, it is remarkable that these drugs uniformly bind to prokaryotic rRNA rather than ribosomal proteins [reviewed by Wilson (51)]. Furthermore, while the target engagement, effectiveness and tolerability of antibiotics is well established, as a class it is noteworthy that the affinity and selectivity of antibiotics for binding to bacterial rRNA, as opposed to off-target sequences in the host transcriptome, is quite modest. These and other observations have fuelled interest in developing novel compounds that target cellular or viral messenger or noncoding RNAs. However, the feasibility of targeting RNAs that are much less abundant and less conformationally constrained than rRNA remains uncertain, and the optimal methods for selecting targets and screening ligands have not been determined.

In this context, DM1 presents an attractive paradigm for RNA targeting. First, the biologic rationale is solid because the mutant *DMPK* mRNA is directly pathogenic, due to the presence of an expanded repeat (52). Second, the CUG^exp^ tract forms a distinctive secondary structure, described as a ‘slippery’ hairpin (53–55). ‘Slippery’ denotes a tendency for U·U mismatches to promote ‘breathing’ or realignment of strands in the hairpin stem. The long r(CUG)_n_ hairpins are stable *in vitro* [*T*_m_ ∼75°C (54)] and presumably also in cells. Third, the mean CTG expansion size in muscle tissue of DM1 patients is 4400 repeats (18) and the repeat tract is fully transcribed (13,14). Thus, while the *DMPK* transcripts are not highly abundant [∼1 per 10 000 mRNAs in muscle (56)], each repeat tract in mutant mRNA presents a huge capacity for protein or ligand binding. Based on observed packing density of one MBNL1 protein per four CUG repeats (57) we estimate that, on average, each mutant transcript may present ∼1000 MBNL1 binding sites. Fourth, the mutant transcripts are concentrated in one or a few foci in muscle nuclei, occupying a tiny fraction of the nuclear volume. In terms of poly-(CUG) binding proteins, the combination of high multivalency and localization in foci is likely to drive strong sequestration and functional inactivation of MBNL1 (15,16,18,58). In terms of small molecules, the same circumstance may drive high levels of ligand binding (59,60). Such an effect may explain why lomofungin and dilomofungin, without further chemical optimization, are exhibiting bioactivity in CUG^exp^-expressing cells, despite target affinities and selectivities that are not highly robust. Fifth, recent evidence suggests that many splicing defects and symptoms of DM1 are triggered when the last remaining fraction of MBNL protein is titrated from the nucleoplasm and sequestered in foci (18,61), which implies that partial release of MBNL protein may be all that is required to improve splicing regulation. And sixth, experience with the CAG25 morpholino, whether by local or systemic administration in transgenic mice, has provided proof of concept that inhibitors of MBNL-CUG^exp^ binding can produce rapid correction of splicing defects and phenotypes *in vivo* (30,62). Since the intracellular delivery of CAG25 remains challenging, the rationale to develop small molecules having similar effects is strong.

Previous studies have used unbiased screens to identify compounds with anti-DM1 activity. A pilot screen (13 000 compounds) employed a cell-based assay to identify molecules that modulate a single DM1-affected splice event (63). The reproducibility of the assay was low (30%) and the hits were not pursued. Ketley *et al.* used imaging to perform a cell-based screen (16 000 compounds) for molecules that inhibited the formation of nuclear CUG^exp^ foci (64). The active compounds showed a reproducible reduction of signal intensity in foci, but, in contrast to CAG25, the nuclear export of CUG^exp^ RNA was not enhanced. These compounds are valuable tools for investigating the still unknown mechanism for localization of CUG^exp^ transcripts in foci.

In contrast, our study is the first biochemical screen for inhibitors of a deleterious RNA–protein interaction in DM1 or other tandem repeat expansion diseases. In principle, a screening effort to inhibit RNA:protein recognition may have the same conceptual limitations as protein–protein binding, namely, the challenge of finding a low molecular weight ligand that blocks a macromolecular interaction involving a large binding interface. However, previous screens for protein:RNA binding inhibitors, and particularly those involving structured RNA, have shown some success, at least in terms of finding *bona fide* inhibitors of *in vitro* binding (65,66). The current study and our separate publication reporting activities for two compounds that showed lower activity in the initial screen [compounds C and I in Supplementary Figure S1 (67)] support the feasibility of using high-throughput methods to find ligands that inhibit protein recognition of repetitive RNA. It remains to be determined whether the number of hits and activity of the candidates can be improved by using larger screens or custom libraries that are specifically designed for targeting nucleic acids.

In our initial characterization, we found that lomofungin exhibits many of the characteristics that would be desired for a small molecule modulator of RNA toxicity. This natural product binds to 5′CNG/3′GNC internal loops with a pyrimidine mismatch *in vitro* and inhibits the binding of (CUG)_12_ to MBNL1. In CUG^exp^-expressing cells it increases the diffuse nucleoplasmic localization and splicing regulatory activity of MBNL1. Further work is needed to determine whether it acts as an intercalator or groove binder, and whether it is possible to enhance its activity and reduce its toxicity by chemical modification or alternative modes of multimerization. Although lomofungin was isolated and characterized as a natural antimicrobial agent in the 1960s, and shown to impact transcription and translation in yeast at high concentration, its precise intracellular targets were never determined, and it is unclear how the RNA binding properties we have uncovered are related to the previous work.

It is noteworthy that phenolic compounds such as lomofungin may have tendencies to produce non-specific aggregation and false positive results in high-throughput screens (68). However, aggregation effects are unlikely to account for the lomofungin activity in our screening assays. (1) The MLSMR library was previously screened for aggregation-induced inhibitors and lomofungin tested negative (PubChem AID 485341) (38). (2) Detergent (0.05% Treen-20) was included in our assays to reduce aggregation effects (69). (3) If aggregation was the mechanism, then inhibition of protein–peptide interaction would be expected in the counter screen. This was not observed. (4) Out of 566 assays in PubChem that included lomofungin, only four showed activity with sub-micromolar potency. Among these, the potency of lomofungin in our screen was greater than any previous assay. These data, along with the bioactivity in CUG^exp^-expressing cells, argue against non-specific signal quenching in our screening assays.

At the time when dimerization was recognized it was no longer possible for us to determine the exact ratio of lomofungin monomer and dimer in the stock that was used in the primary screen. However, comparisons across assays suggest that material in the primary screen was a mixture of both (IC_50_ value of 291 nM in the primary AlphaScreen versus 717 nM and 42 nM in subsequent assays for pure monomer and fully converted dimer, respectively). Regardless, the activity of monomer was sufficient to secure a place for lomofungin among the top 10 hits in the screen, and it appears therefore that discovery of a dimer with greater inhibitory activity was largely accidental. This difference between binding inhibition by monomer and dimer is perfectly aligned with previous findings that assembly of r(CUG)_n_-binding monomers into dimers or higher order multimers produced major improvements of (CUG)_n_ affinity, selectivity and MBNL1 binding inhibition (23,26,28,33,34,70). However, in previous studies the monomers were coupled using an intersubunit spacer, and empiric optimization of the spacer length and composition in some cases produced a > 100-fold improvement of the inhibition potency. By comparison, the monomers in dilomofungin are directly coupled, producing a 17-fold improvement of potency, and resulting in an IC_50_ that was 8-fold lower than previously reported dimers. It will be interesting to determine whether other coupling arrangements of lomofungin monomers can further modulate its inhibitory properties.

Dilomofungin was more potent than other compounds in the screening set yet it exhibited troubling cellular effects. Our observation that a strong inhibitor caused marked cellular accumulation of CUG^exp^ RNA was unexpected. We are not aware of previous instances in which screens for modulators of RNA–protein binding have identified compounds with dramatic effects on RNA turnover. The mechanism for target stabilization in CUG^exp^-expressing cells is unknown. Conceivably, it may reflect an unintended consequence of the binding inhibition. Using transcriptome-wide analyses, previous studies have shown that knockdown of MBNL1 led to increased expression of mRNAs that have MBNL1 binding sites in the 3′ UTR, suggesting that MBNL1 binding has a net destabilizing effect (19,71). When averaged across the entire spectrum of MBNL1 targets the effect was small (<5% change of the mean expression level) (19). However, certain endogenous transcripts with multiple MBNL1 binding sites in the 3′ UTR showed a 2-fold increase of mRNA half-life after MBNL1 knockdown (71). For transcripts with expanded CUG repeats and many binding sites in the 3′ UTR, it seems possible that effects of MBNL1 on mRNA decay are more pronounced. If correct, the stabilization of *fluc*-CUG^exp^ by dilomofungin may simply reflect highly effective inhibition of MBNL1 binding. By this ‘diminishing returns’ model, the process of optimizing ligands for binding inhibition may inevitably lead at some point to transcript accumulation and reduced efficacy, which is essentially what we observed with the dimerization of lomofungin. However, it is unclear whether the previous observations of MBNL1 effects on mRNA turnover in the cytoplasm would apply to nuclear retained transcripts, where the pathways for mRNA degradation are less defined. Also, we observed that CAG25 morpholino has the opposite effect of reducing levels of CUG^exp^ accumulation (30), though it is possible that antisense binding may have distinct effects on metabolic fate. An alternative possibility is that target stabilization may prove to be a unique property of dilomofungin, related to specific effects on exonuclease processivity or other aspects of nuclear decay, that are avoidable for other inhibitors with similar potency. Whatever mechanisms may apply, our results suggest that future efforts to develop binding inhibitors for toxic RNA, whether by chemical screens or structural predictions, should assess the impact on RNA turnover at an early stage.

## SUPPLEMENTARY DATA

Supplementary Data are available at NAR Online.

## FUNDING

University of Rochester Wellstone Muscular Dystrophy Cooperative Research Center [U54NS048843]; Marigold Foundation postdoctoral fellowship (to C.Z.C.); Intramural Research Programs of the National Center for Advancing Translational Sciences; National Institutes of Health [AR049077, N2058345, 5R21NS071023 to B.L.M., 1R01GM100788 to B.L.M. and 1R01GM079235 to M.D.D.]. Source of open access funding: National Institutes of Health and institutional funds.

*Conflict of interest statement*. None declared.

## Supplementary Material

SUPPLEMENTARY DATA
