# Supplementary material for: Lomofungin and dilomofungin: inhibitors of MBNL1-CUG RNA binding with distinct cellular effects
Source: Nucleic Acids Res. 2014 May 5;42(10):6591–602. doi: 10.1093/nar/gku275 (PMC4041448; doi:10.1093/nar/gku275)
Supplement: SUPPLEMENTARY DATA [file supp_42_10_6591__index.html]

Lomofungin and dilomofungin: inhibitors of MBNL1-CUG RNA binding with distinct cellular effects — SUPPLEMENTARY DATA 

# Lomofungin and dilomofungin: inhibitors of MBNL1-CUG RNA binding with distinct cellular effects

## SUPPLEMENTARY DATA

**Files in this Data Supplement:**

- SUPPLEMENTARY DATA
- SUPPLEMENTARY DATA
