# Supplementary material for: Lomofungin and dilomofungin: inhibitors of MBNL1-CUG RNA binding with distinct cellular effects
Source: Nucleic Acids Res. 2014 May 5;42(10):6591–602. doi: 10.1093/nar/gku275 (PMC4041448; doi:10.1093/nar/gku275)
Supplement: SUPPLEMENTARY DATA [file supp_gku275_nar-01336-y-2013-File012.pdf]

[Bound Lomofungin] (nM)	Internal loops or MBNL1	Pairwise fold binding difference							Pairwise *P*-values for binding difference						
		1x2 CUG	2x2 CUG	3x2 CUG	4x2 CUG	5x2 CUG	6x2 CUG	MBNL1	1x2 CUG	2x2 CUG	3x2 CUG	4x2 CUG	5x2 CUG	6x2 CUG	MBNL1
1027 ± 157	**1x2 CUG**	1.00	1.52	1.98	2.26	2.49	2.76	-2.18	NA	0.0048	0.0015	0.0021	0.0013	0.0009	0.0049
1559 ± 231	**2X2 CUG**	-1.52	1.00	1.30	1.49	1.64	1.82	-3.32	0.0048	NA	0.0407	0.0221	0.0104	0.0055	0.0017
2028 ± 375	**3X2 CUG**	-1.98	-1.30	1.00	1.15	1.26	1.40	-4.32	0.0015	0.0407	NA	0.2078	0.093	0.0401	0.0028
2323 ± 543	**4X2 CUG**	-2.26	-1.49	-1.15	1.00	1.10	1.22	-4.94	0.0021	0.0221	0.2078	NA	0.2923	0.1418	0.0056
2558 ± 585	**5X2 CUG**	-2.49	-1.64	-1.26	-1.10	1.00	1.11	-5.44	0.0013	0.0104	0.093	0.2923	NA	0.2815	0.0048
2830 ± 647	**6X2 CUG**	-2.76	-1.82	-1.40	-1.22	-1.11	1.00	-6.02	0.0009	0.0055	0.0401	0.1418	0.2815	NA	0.0044
470 ± 8	**MBNL1**	2.18	3.32	4.32	4.94	5.44	6.02	1.00	0.0049	0.0017	0.0028	0.0056	0.0048	0.0044	NA

Supplemental Table 2. Quantitative pairwise comparisons of lomofungin binding to MBNL1 or RNA hairpins with the indicated number of 5'CUG/3'GUC internal loops. Bound lomofungin concentrations are presented with 95% confidence intervals. Yellow highlighted cells indicate significant pairwise differences based on *t*-tests of the bound concentrations (α threshold was FDR adjusted for multiple testing correction).

[Bound Dilomofungin] (nM)	Internal loops or MBNL1	Pairwise fold binding difference							Pairwise *P*-values for binding difference						
		1x2 CUG	2x2 CUG	3x2 CUG	4x2 CUG	5x2 CUG	6x2 CUG	MBNL1	1x2 CUG	2x2 CUG	3x2 CUG	4x2 CUG	5x2 CUG	6x2 CUG	MBNL1
934 ± 149	**1x2 CUG**	1.00	1.50	2.10	2.35	2.50	2.75	-1.60	NA	0.0083	0.0004	0.0002	0.0002	7E-05	0.023
1402 ± 95	**2X2 CUG**	-1.50	1.00	1.40	1.57	1.66	1.84	-2.40	0.0083	NA	0.0049	0.0051	0.0032	0.0012	0.0056
1965 ± 56	**3X2 CUG**	-2.10	-1.40	1.00	1.12	1.19	1.31	-3.37	0.0004	0.0049	NA	0.0412	0.0142	0.0022	0.0016
2196 ± 127	**4X2 CUG**	-2.35	-1.57	-1.12	1.00	1.06	1.17	-3.76	0.0002	0.0051	0.0412	NA	0.1254	0.0168	0.0018
2334 ± 111	**5X2 CUG**	-2.50	-1.66	-1.19	-1.06	1.00	1.10	-4.00	0.0002	0.0032	0.0142	0.1254	NA	0.0319	0.0014
2573 ± 56	**6X2 CUG**	-2.75	-1.84	-1.31	-1.17	-1.10	1.00	-4.41	7E-05	0.0012	0.0022	0.0168	0.0319	NA	0.0008
583 ± 143	**MBNL1**	1.60	2.40	3.37	3.76	4.00	4.41	1.00	0.023	0.0056	0.0016	0.0018	0.0014	0.0008	NA

Supplemental Table 3. Quantitative pairwise comparisons of dilomofungin binding to MBNL1 or RNA hairpins with the indicated number of 5'CUG/3'GUC internal loops. Bound dilomofungin concentrations are presented with 95% confidence intervals. Yellow highlighted cells indicate significant pairwise differences based on *t*-tests of the bound concentrations (α threshold was FDR adjusted for multiple testing correction).

[Bound Lomofungin] (nM)	Internal loop mismatch	Pairwise fold binding difference											Pairwise *P*-values for binding difference										
		U-U	C-C	A-A	G-G	U-C	A-C	C-U	G-A	C-A	A-G	No loop	U-U	C-C	A-A	G-G	U-C	A-C	C-U	G-A	C-A	A-G	No loop
1027 ± 157	**U-U**	1.00	-1.76	-2.52	-3.20	-1.76	-1.62	1.06	-3.43	-3.56	-3.01	-2.71	NA	0.0114	0.0035	0.0021	0.0142	0.0192	0.3668	0.0020	0.0018	0.0029	0.0030
582 ± 95	**C-C**	1.76	1.00	-1.43	-1.81	1.00	1.09	1.87	-1.94	-2.02	-1.70	-1.53	0.0114	NA	0.0456	0.0195	0.4946	0.3085	0.0595	0.0196	0.0132	0.0531	0.0369
407 ± 58	**A-A**	2.52	1.43	1.00	-1.27	1.43	1.56	2.67	-1.36	-1.41	-1.19	-1.07	0.0035	0.0456	NA	0.0726	0.1027	0.0544	0.0343	0.0648	0.0292	0.2402	0.2950
321 ± 43	**G-G**	3.20	1.81	1.27	1.00	1.82	1.98	3.38	-1.07	-1.11	1.06	1.18	0.0021	0.0195	0.0726	NA	0.0532	0.0293	0.0274	0.3121	0.1401	0.4043	0.1401
583 ± 177	**U-C**	1.76	-1.00	-1.43	-1.82	1.00	1.09	1.86	-1.95	-2.02	-1.71	-1.54	0.0142	0.4946	0.1027	0.0532	NA	0.3531	0.0674	0.0487	0.0414	0.0846	0.0838
635 ± 150	**A-C**	1.62	-1.09	-1.56	-1.98	-1.09	1.00	1.71	-2.12	-2.20	-1.86	-1.67	0.0192	0.3085	0.0544	0.0293	0.3531	NA	0.0765	0.0278	0.0228	0.0529	0.0457
1087 ± 364	**C-U**	-1.06	-1.87	-2.67	-3.38	-1.86	-1.71	1.00	-3.63	-3.77	-3.18	-2.87	0.3668	0.0595	0.0343	0.0274	0.0674	0.0765	NA	0.0263	0.0250	0.0321	0.0321
300 ± 61	**G-A**	3.43	1.94	1.36	1.07	1.95	2.12	3.63	1.00	-1.04	1.14	1.27	0.0020	0.0196	0.0648	0.3121	0.0487	0.0278	0.0263	NA	0.3811	0.3193	0.1104
289 ± 9	**C-A**	3.56	2.02	1.41	1.11	2.02	2.20	3.77	1.04	1.00	1.18	1.31	0.0018	0.0132	0.0292	0.1401	0.0414	0.0228	0.0250	0.3811	NA	0.2648	0.0559
342 ± 138	**A-G**	3.01	1.70	1.19	-1.06	1.71	1.86	3.18	-1.14	-1.18	1.00	1.11	0.0029	0.0531	0.2402	0.4043	0.0846	0.0529	0.0321	0.3193	0.2648	NA	0.3383
379 ± 64	**No loop**	2.71	1.53	1.07	-1.18	1.54	1.67	2.87	-1.27	-1.31	-1.11	1.00	0.0030	0.0369	0.2950	0.1401	0.0838	0.0457	0.0321	0.1104	0.0559	0.3383	NA

Supplemental Table 4. Quantitative pairwise comparisons of lomofungin binding to RNA with the indicated mismatch in an internal loop (or no loop). Bound lomofungin concentrations are presented with 95% confidence intervals. Yellow highlighted cells indicate significant pairwise differences in lomofungin binding based on *t*-tests of bound concentrations (α threshold was FDR adjusted for multiple testing correction).

Bound Dilomofungin (nM)	Internal loop mismatch	Pairwise fold binding difference											Pairwise *P*-values for binding difference										
		U-U	C-C	A-A	G-G	U-C	A-C	C-U	G-A	C-A	A-G	No loop	U-U	C-C	A-A	G-G	U-C	A-C	C-U	G-A	C-A	A-G	No loop
934 ± 149	U-U	1.00	-4.06	-1.27	-3.89	-5.41	-8.51	-6.51	-3.49	-3.86	-12.00	-4.09	NA	0.0019	0.0909	0.0021	0.0031	0.001	0.0012	0.0022	0.0019	0.0009	0.002
230 ± 66	C-C	4.06	1.00	3.19	1.04	-1.33	-2.10	-1.60	1.16	1.05	-2.96	-1.01	0.0019	NA	0.0136	0.4359	0.3686	0.0415	0.0775	0.194	0.3988	0.0283	0.4908
734 ± 108	A-A	1.27	-3.19	1.00	-3.06	-4.25	-6.69	-5.11	-2.75	-3.03	-9.43	-3.21	0.0909	0.0136	NA	0.0156	0.0383	0.0079	0.0089	0.0134	0.0131	0.0072	0.017
240 ± 86	G-G	3.89	-1.04	3.06	1.00	-1.39	-2.19	-1.67	1.11	1.01	-3.09	-1.05	0.0021	0.4359	0.0156	NA	0.3499	0.0534	0.0904	0.301	0.4867	0.0374	0.4415
173 ± 201	U-C	5.41	1.33	4.25	1.39	1.00	-1.57	-1.20	1.55	1.40	-2.22	1.32	0.0031	0.3686	0.0383	0.3499	NA	0.3547	0.4308	0.2909	0.3417	0.292	0.3772
110 ± 29	A-C	8.51	2.10	6.69	2.19	1.57	1.00	1.31	2.44	2.21	-1.41	2.08	0.001	0.0415	0.0079	0.0534	0.3547	NA	0.1477	0.0047	0.0206	0.1468	0.0883
144 ± 26	C-U	6.51	1.60	5.11	1.67	1.20	-1.31	1.00	1.86	1.69	-1.84	1.59	0.0012	0.0775	0.0089	0.0904	0.4308	0.1477	NA	0.0116	0.0408	0.0609	0.1433
267 ± 7	G-A	3.49	-1.16	2.75	-1.11	-1.55	-2.44	-1.86	1.00	-1.10	-3.43	-1.17	0.0022	0.194	0.0134	0.301	0.2909	0.0047	0.0116	NA	0.1986	0.0041	0.2793
242 ± 32	C-A	3.86	-1.05	3.03	-1.01	-1.40	-2.21	-1.69	1.10	1.00	-3.11	-1.06	0.0019	0.3988	0.0131	0.4867	0.3417	0.0206	0.0408	0.1986	NA	0.0147	0.4209
77.8 ± 33	A-G	12.00	2.96	9.43	3.09	2.22	1.41	1.84	3.43	3.11	1.00	2.93	0.0009	0.0283	0.0072	0.0374	0.292	0.1468	0.0609	0.0041	0.0147	NA	0.0615
228 ± 77	No loop	4.09	1.01	3.21	1.05	-1.32	-2.08	-1.59	1.17	1.06	-2.93	1.00	0.002	0.4908	0.017	0.4415	0.3772	0.0883	0.1433	0.2793	0.4209	0.0615	NA

Supplemental Table 5. Quantitative pairwise comparisons of dilomofungin binding to RNA with the indicated mismatch in an internal loop (or no loop). Bound dilomofungin concentrations are presented with 95% confidence intervals. Yellow highlighted cells indicate significant pairwise differences in dilomofungin binding based on *t*-tests of bound concentrations (α threshold was FDR adjusted for multiple testing correction).

Target gene	Primer name	Primer sequence
Mapkapk5	JHO191	GTG CGC CTG CAC ATG ATG TG
	JHO192	TTG AGG TCT CTG TGC GCA ATG
Arid2	JHO193	TCG CTT GTC AGT GGC TAA ATG C
	JHO194	GGA CAA CAG GAG TGG AAA TTG GC
Nr3c1	JHO195	TCC CTT TCT CAG CAG CAG GAT C
	JHO196	CGG AGG AGA ACT CAC ATC TGG TC
rRNA 5' ETS	mrRNAETS-f-B	GTG ACT TTG CTG CGT GTC AGA C
	mrRNAETS-r-B	CCA CAG ACA GGA GTG AAG TAC TCG
Mapkap1	Mapkap1 F	ACC CTG TTA CGA ATC AGA AAG CCA
	Mapkap1 R	TCT GGA AGC TGA AGC TTG TTC G

Supplemental Table 6. PCR primers used in semi-quantitative RT-PCR

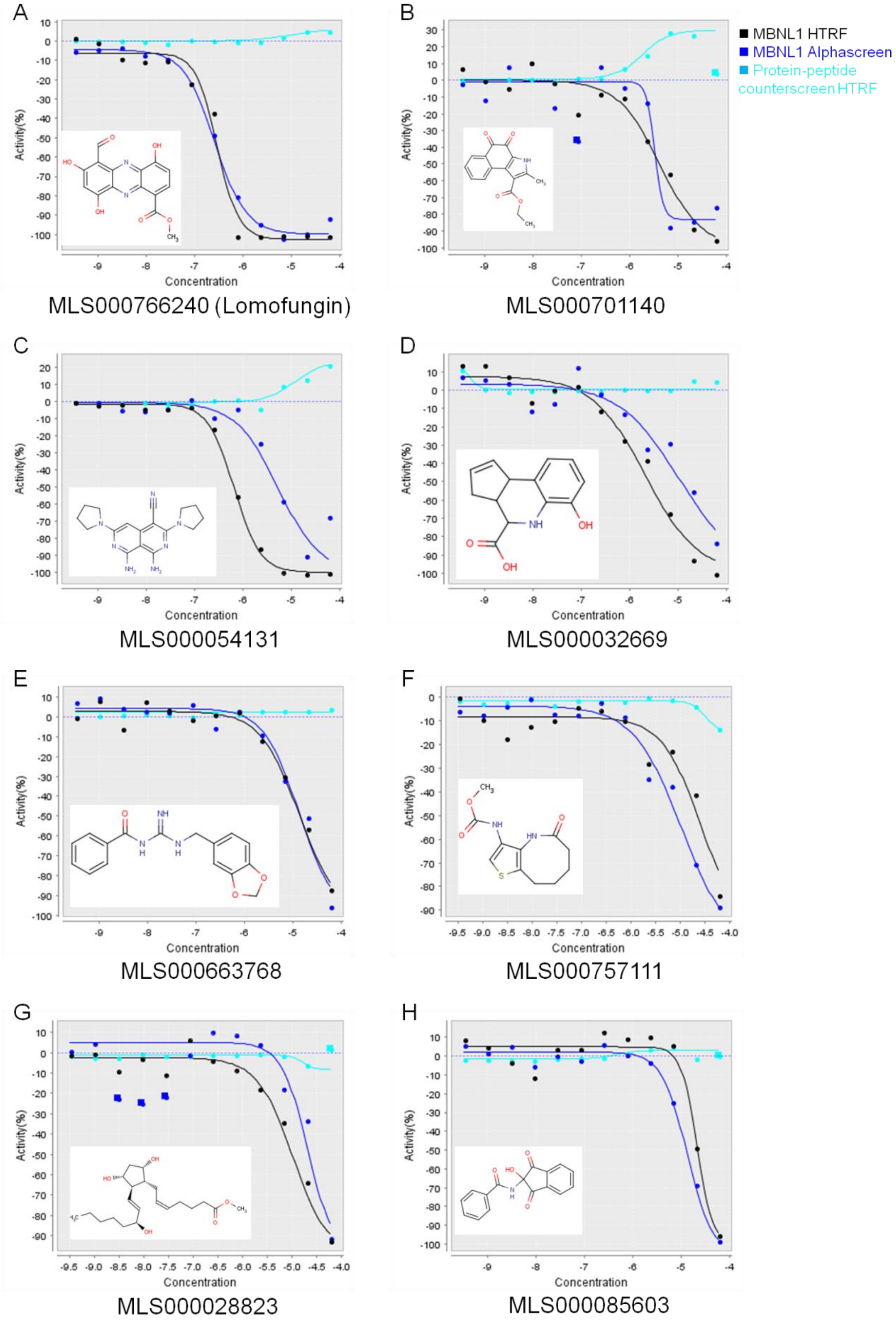
A
B
MBNL1 HTRF
MBNL1 Alphascreen
Protein-peptide counterscreen HTRF
Activity(%)
Concentration
MLS000766240 (Lomofungin)
MLS000701140
C
D
MLS000054131
MLS000032669
E
F
MLS000663768
MLS000757111
G
H
MLS000028823
MLS000085603

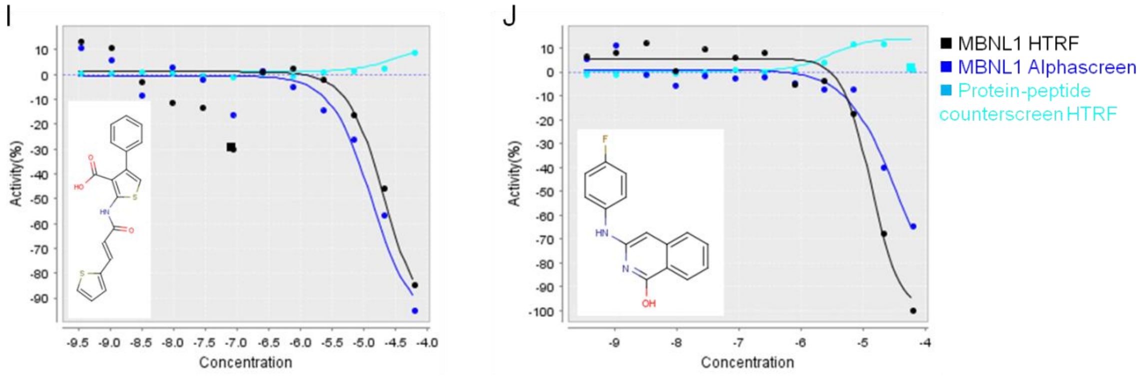

MLS000773385 MLS000565365

Supplemental Figure 1. Top ten confirmed inhibitors of MBNL1-$(CUG)_{12}$ RNA binding from the high throughput screen. Results of the HTRF, AlphaScreen and counter screen involving a known protein-peptide interaction (negative control for nonspecific signal quenching) are presented with $\log_{10}$(M) compound concentrations. The compounds are presented in rank order by IC_{50}. Inhibition by lomofungin (A) is at least 2-fold more potent than the next best compound (B).

A

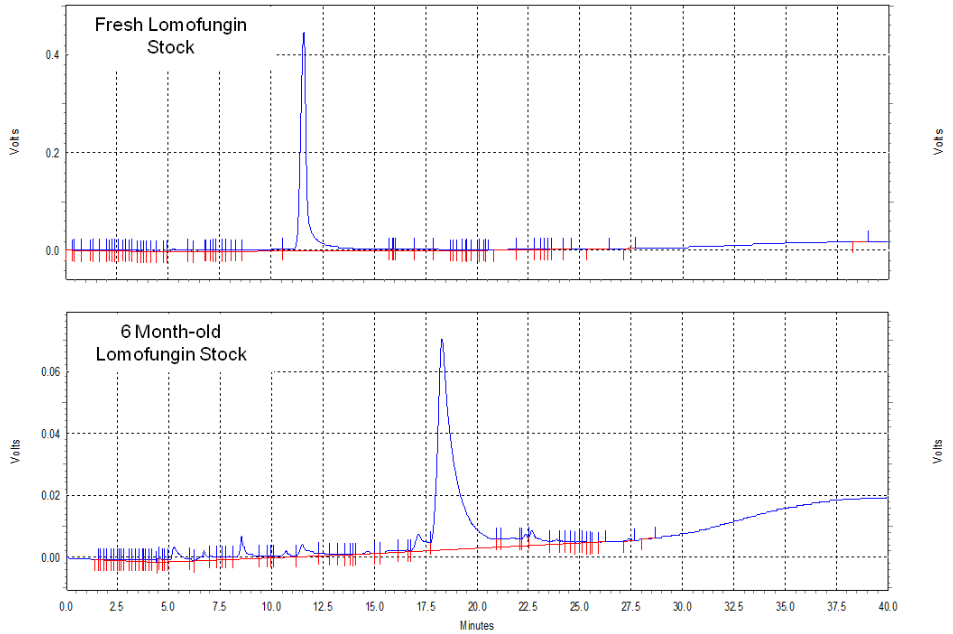

B

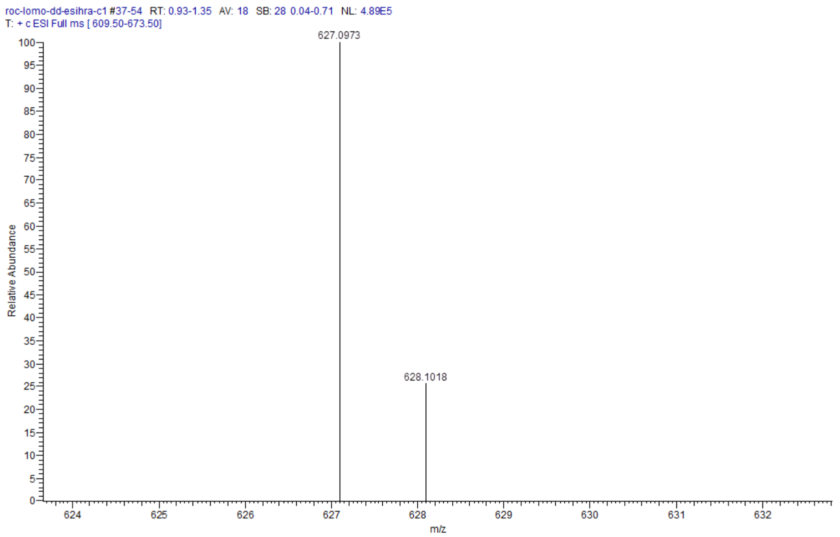

Supplemental Figure 2. Lomofungin dimerizes in DMSO. (A) Reverse phase HPLC of freshly prepared lomofungin in DMSO versus a 10 mM stock in DMSO stored at 4 °C for 6 months. (B) High resolution mass spectrometry of material in the old lomofungin stock indicates m/z of 627.0973, corresponding to the predicted m/z of $C_{30}H_{19}N_4O_{12}$ $[M+H]^+$ (627.0994), which is equivalent to 2X lomofungin, less 2 AMU.

A

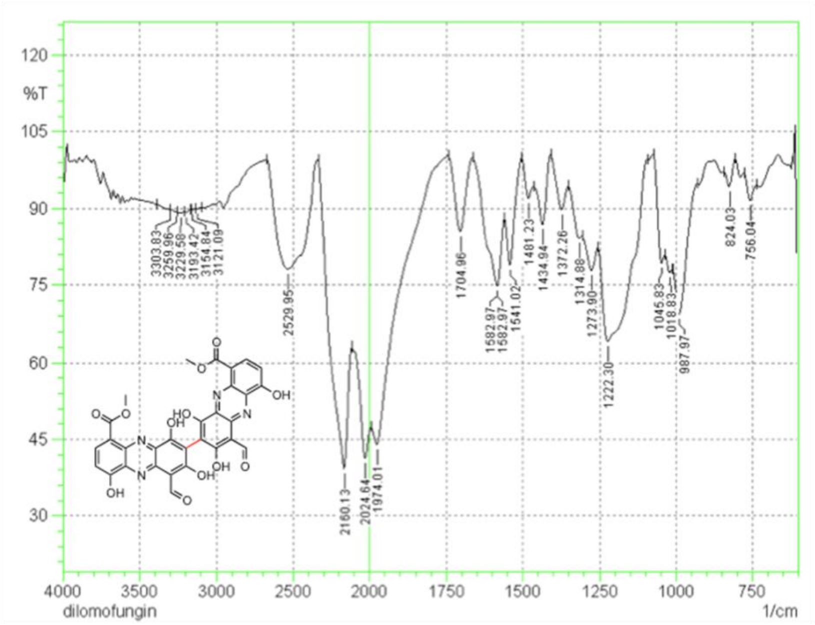
%T
120
105
90
75
60
45
30
3303.83
3259.96
3229.58
3193.42
3154.84
3121.09
2529.95
2160.13
2024.64
1974.01
1704.96
1582.97
1582.97
1541.02
1481.23
1434.94
1372.26
1314.88
1273.90
1222.30
1045.83
1018.83
987.97
824.03
756.04
4000 3500 3000 2500 2000 1750 1500 1250 1000 750
dilomofungin
1/cm

B

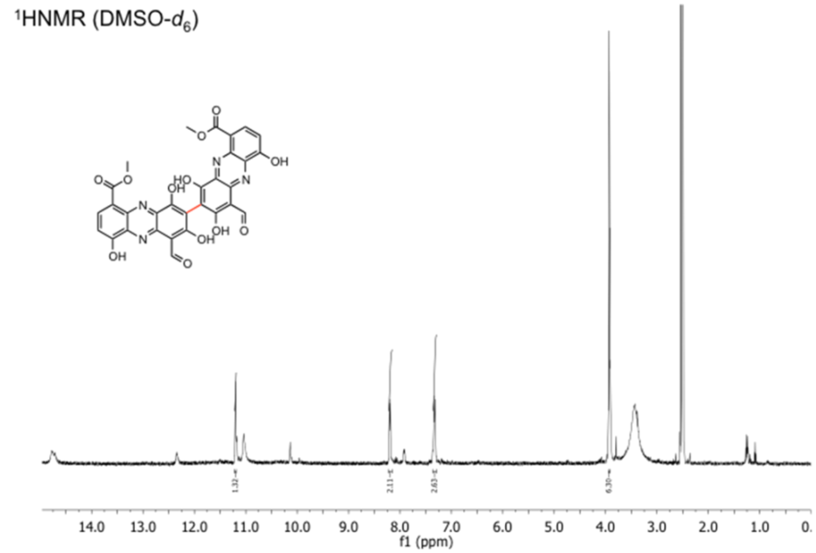
^{1}HNMR (DMSO-d_6)
14.0 13.0 12.0 11.0 10.0 9.0 8.0 7.0 6.0 5.0 4.0 3.0 2.0 1.0 0.
f1 (ppm)

C

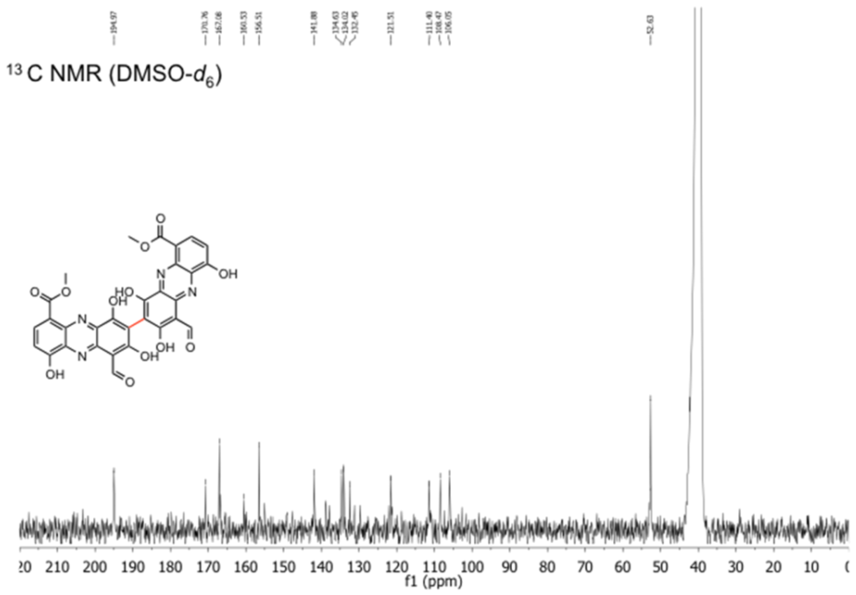

D

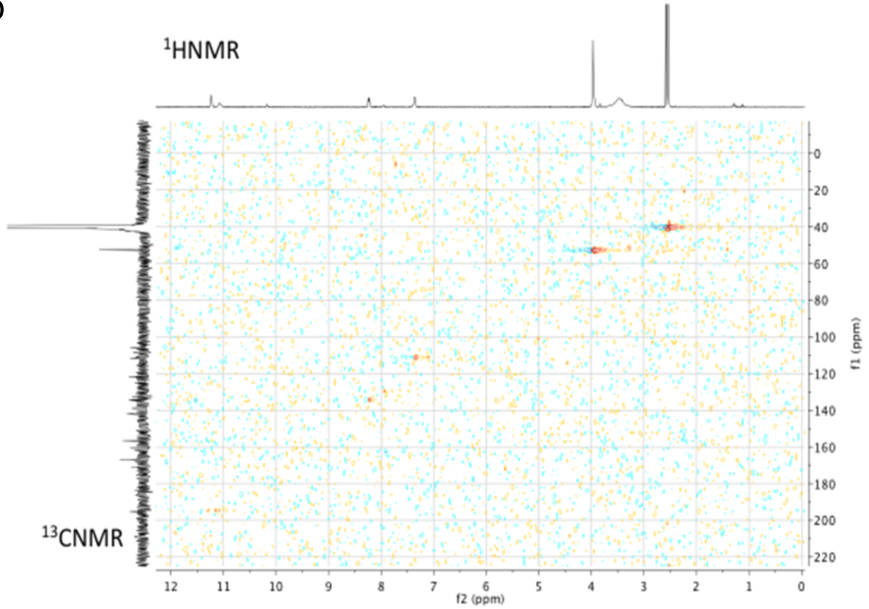

Supplemental Figure 3. Spectral analyses confirm the structure of dilomofungin. (A) Dilomofungin FTIR (neat): 3303.83, 3259.96, 3229.58, 3193.42, 3154.84, 3121.09, 2529.95, 2160.13, 2024.64, 1974.01, 1704.96, 1582.97, 1582.97, 1541.02, 1481.23, 1434.94, 1372.26, 1314.88, 1273.9, 1222.3, 1045.83, 1018.83, 987.97, 824.03, 756.04. (B) Dilomofungin ^{1}H NMR (500 MHz, DMSO-*d6*) δ 11.20 (s, 2H), 8.20 (d, J = 8.0 Hz, 2H), 7.33 (d, J = 8.0 Hz, 2H), 3.93 (s, 6H). (C) Dilomofungin ^{13}C NMR (125 MHz, DMSO-*d6*) δ 194.97, 170.76, 167.08, 160.53, 156.51, 141.88, 134.63, 134.02, 132.45, 121.51, 111.40, 108.47, 106.05, 52.63. (D) 2D ^{1}H^{13}C HSQC spectrum of dilomofungin.

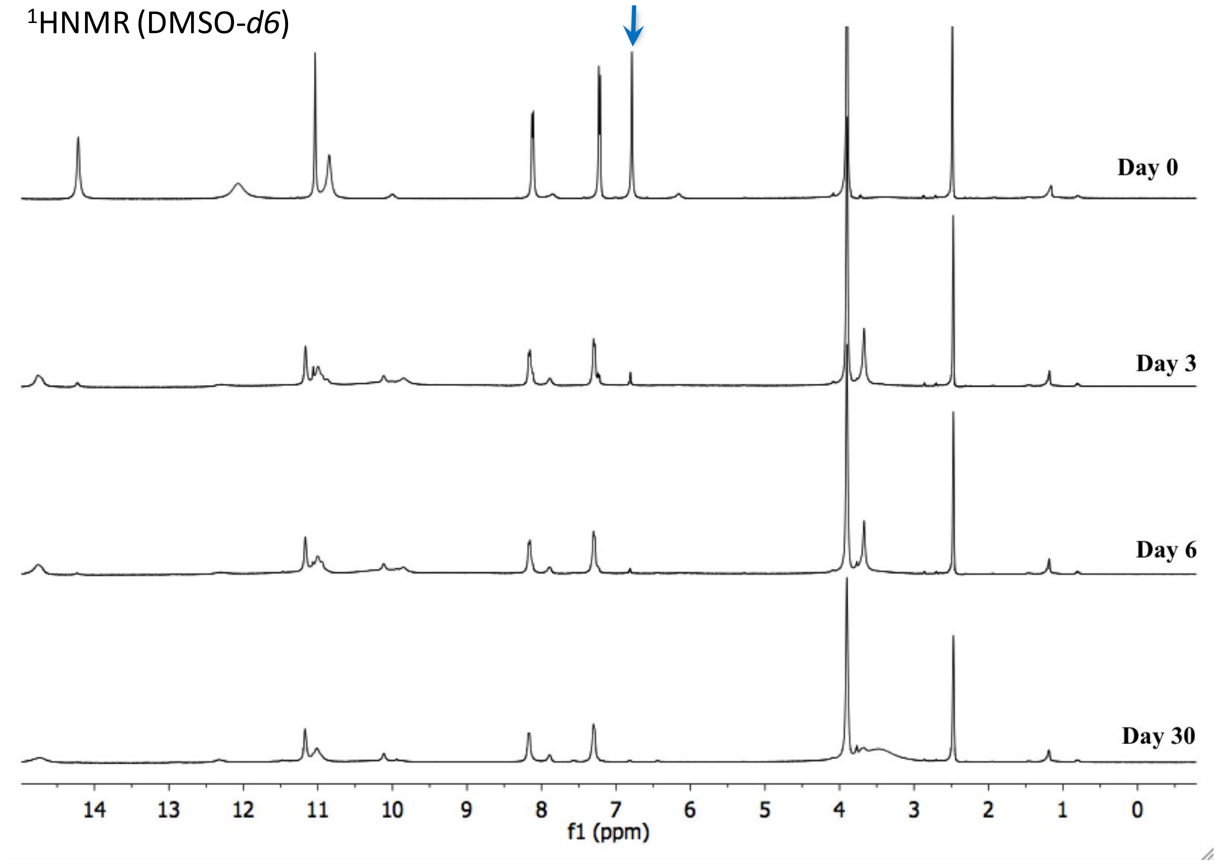

Supplemental Figure 4. Series of proton NMR spectra for lomofungin collected over a period of 30 days in DMSO-d_6 at room temperature. After day 3 the intensity of singlet proton peak at the ortho position (indicated by blue arrow) had diminished significantly and was not observable after day 30.

A

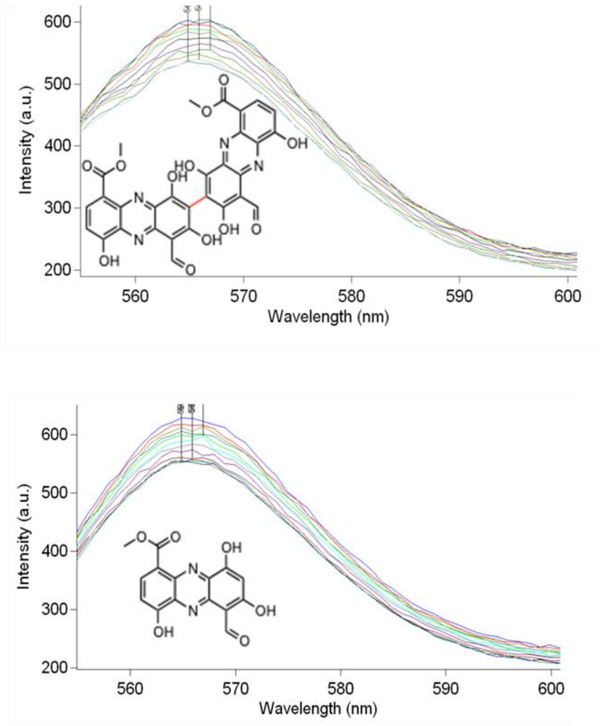

B

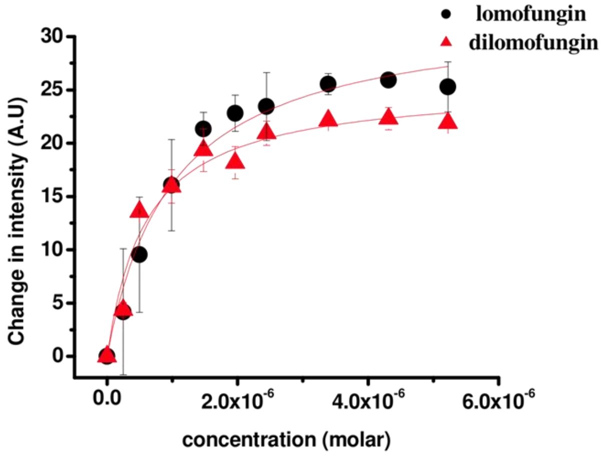

C

Compound	Binding constant, K_D (μM)
**lomofungin**	0.998 ± 0.176
**dilomofungin**	0.606 ± 0.310

Supplemental Figure 5. Dilomofungin binds to CUG repeat RNA with higher affinity than lomofungin. (A) Selected fluorescence titration curves of lomofungin (bottom) and dilomofungin (top) with Cy3-$(CUG)_{10}$ RNA showing dose dependent fluorescence quenching. (B) Change in Cy3 fluorescence intensity versus drug concentration from fluorescence titration experiment. The data were fit to a one binding site equation. (C) The calculated binding constants (K_D ± 1 s.d.) based on the binding curves from the fluorescence titration data.

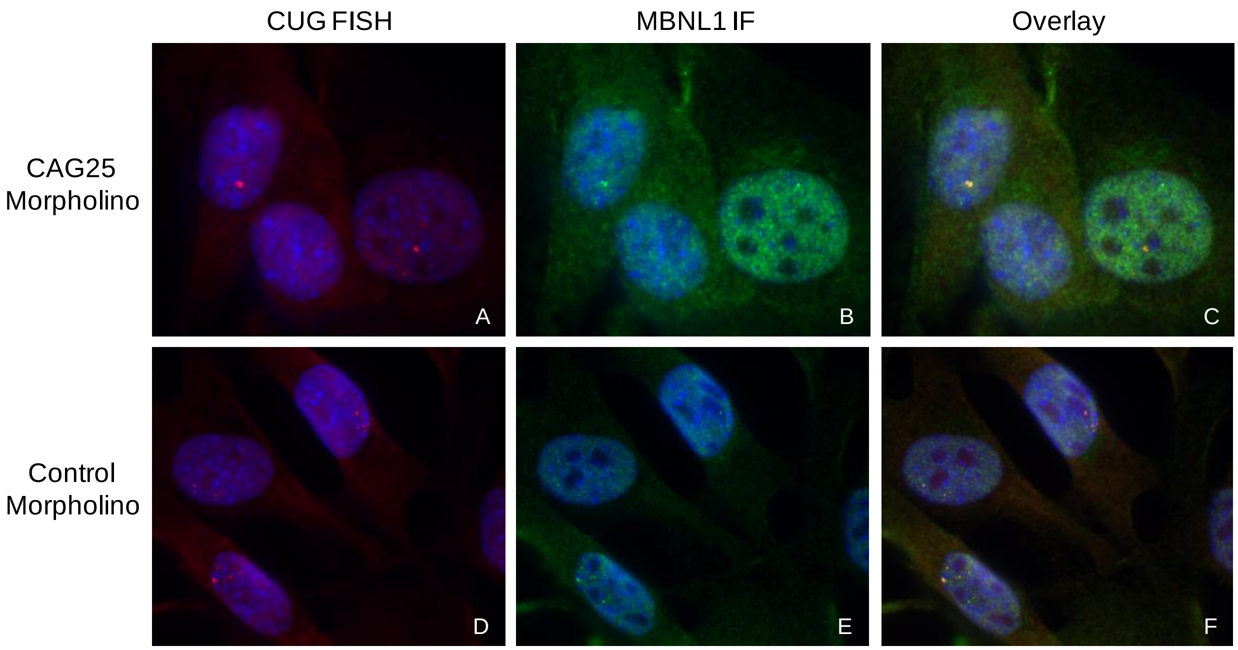

Supplemental Figure 6. Fluorescence *in situ* hybridization (FISH) combined with immunofluorescence (IF) shows that nucleofection of CAG25 produces partial release of MBNL1 from nuclear CUG^{exp} foci in fluc800 cells. (A-C) Fluc800 cells nucleofected with CAG25. (D-F) Fluc800 cells nucleofected with irrelevant antisense morpholino directed against exon 362 of *Titin.* (A, D) FISH for CUG repeat RNA (red), nuclear stain with DAPI (blue). (B,E) IF for MBNL1 (green), nuclear stain with DAPI (blue). (C,F) Overlay of FISH and IF. Fluorescence signals in each column were acquired and displayed with identical exposure and threshold settings.

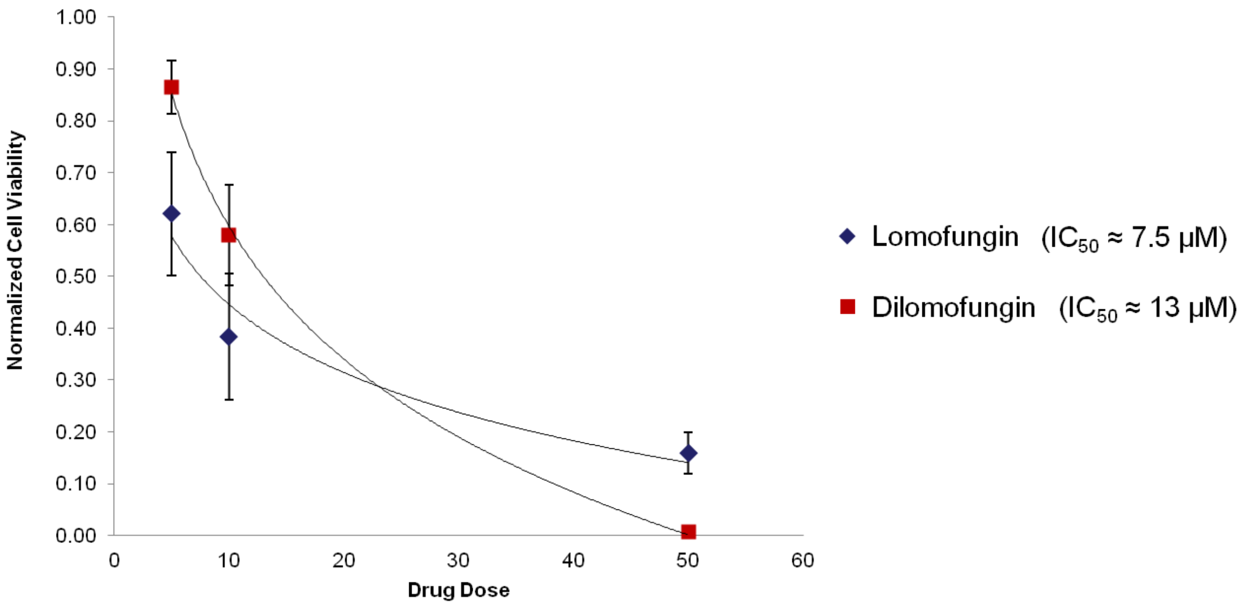

Supplemental Figure 7. Cytotoxicity of lomofungin and dilomofungin in C2C12 cells. Toxicities were assessed by WST-1 assays after 3 days of treatment. Cell viabilities were calculated as the background corrected absorbances at 450 nM – 690 nM, and normalized to 0.1% DMSO treated cells. Curves were fit by logarithmic regression, and approximate toxicity IC_{50} values were calculated from these best fit curve functions. Error bars represent 1 s.d.

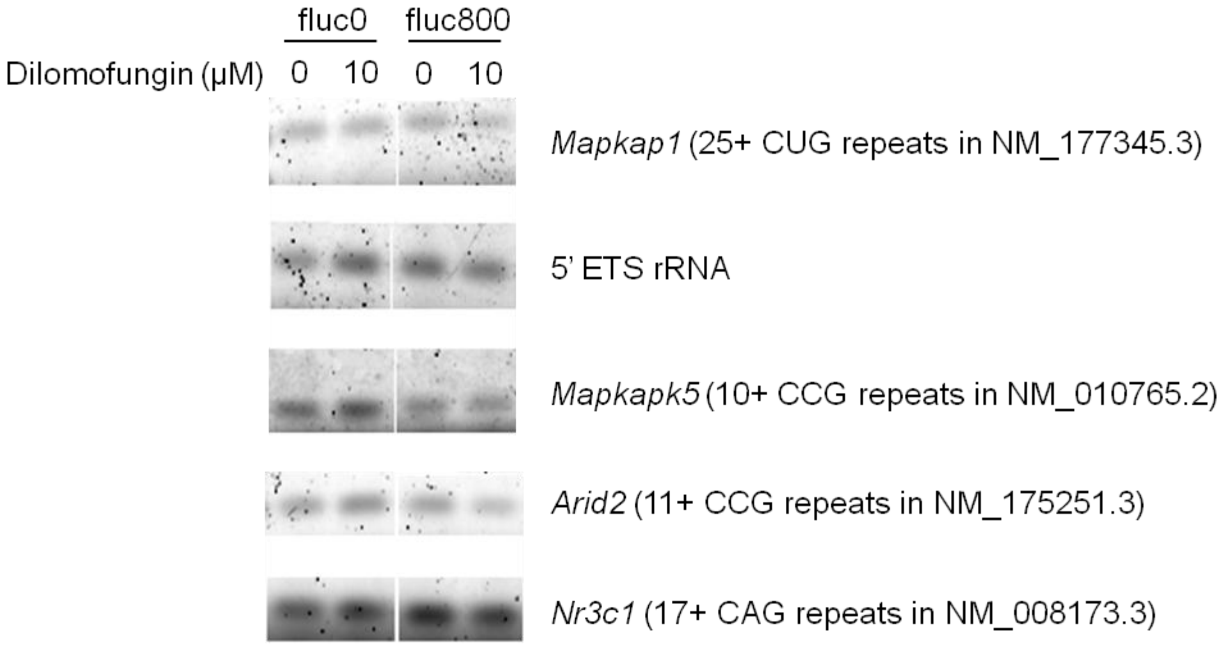

Supplemental Figure 8. Semi-quantitative RT-PCR analysis of the indicated transcripts reveals no additional mRNA accumulation after dilomofungin treatment. Mapkap1, Mapkapk5, Arid2 and Nr3c1 mRNA, and 5' external transcribed spacer (ETS) rRNA levels appear unaffected by dilomofungin treatment in either fluc0 or fluc800 cells. All PCRs were carried out within exponential amplification range. Amplicons were detected by laser scanning of SybrGreen-stained agarose gels.
